# Blockade of SARS-CoV-2 spike protein-mediated cell–cell fusion using COVID-19 convalescent plasma

**DOI:** 10.1038/s41598-021-84840-3

**Published:** 2021-03-10

**Authors:** Ling Wang, Juan Zhao, Lam N. T. Nguyen, James L. Adkins, Madison Schank, Sushant Khanal, Lam N. Nguyen, Xindi Dang, Dechao Cao, Bal Krishna Chand Thakuri, Zeyuan Lu, Jinyu Zhang, Yi Zhang, Xiao Y. Wu, Mohamed El Gazzar, Shunbin Ning, Jonathan P. Moorman, Zhi Q. Yao

**Affiliations:** 1 Center of Excellence for Inflammation, Infectious Disease and Immunity, James H. Quillen College of Medicine, East Tennessee State University, Johnson City, TN 37614 USA; 2 Division of Infectious, Inflammatory and Immunologic Diseases, Department of Internal Medicine, Quillen College of Medicine, ETSU, Johnson City, TN 37614 USA; 3 Hepatitis (HCV/HBV/HIV) Program, Department of Veterans Affairs, James H. Quillen VA Medical Center, Johnson City, TN 37614 USA

**Keywords:** Immunology, Microbiology

## Abstract

The recent COVID-19 pandemic poses a serious threat to global public health, thus there is an urgent need to define the molecular mechanisms involved in SARS-CoV-2 spike (S) protein-mediated virus entry that is essential for preventing and/or treating this emerging infectious disease. In this study, we examined the blocking activity of human COVID-19 convalescent plasma by cell–cell fusion assays using SARS-CoV-2-S-transfected 293 T as effector cells and ACE2-expressing 293 T as target cells. We demonstrate that the SARS-CoV-2 S protein exhibits a very high capacity for membrane fusion and is efficient in mediating virus fusion and entry into target cells. Importantly, we find that COVID-19 convalescent plasma with high titers of IgG neutralizing antibodies can block cell–cell fusion and virus entry by interfering with the SARS-CoV-2-S/ACE2 or SARS-CoV-S/ACE2 interactions. These findings suggest that COVID-19 convalescent plasma may not only inhibit SARS-CoV-2-S but also cross-neutralize SARS-CoV-S-mediated membrane fusion and virus entry, supporting its potential as a preventive and/or therapeutic agent against SARS-CoV-2 as well as other SARS-CoV infections.

## Introduction

Severe Acute Respiratory Syndrome (SARS) triggered the first global alert for coronavirus (CoV) infections in 2003^1,2^. Almost ten years later, a new CoV infection termed the Middle East Respiratory Syndrome (MERS) caused global outbreaks in 2012^3,4^. In late 2019, another novel CoV (SARS-CoV-2) was identified as the causative agent of a global pandemic of viral pneumonia, named by the World Health Organization (WHO) as novel coronavirus infectious disease-2019 (COVID-19)^5^. Although the mortality rate due to COVID-19 is relatively lower, the number of deaths has already surpassed those of SARS and MERS combined, owing to the extremely high transmissibility of SARS-CoV-2^6–9^. As of November 20, 2020, there have been 56,623,643 confirmed cases of COVID-19 and 1,355,963 deaths reported worldwide by the WHO with 11,413,788 confirmed cases and 248,571 deaths in the United States alone^5^. This pandemic nearly shutdown social and economic activities and poses a serious threat to global public health, calling for prompt development of anti-COVID-19 therapeutics and prophylactics for treatment and prevention of future outbreaks.

To combat the COVID-19 pandemic, there is an urgent need to define the molecular mechanism involved in SARS-CoV-2 spike (S) protein-mediated cell–cell fusion that is essential for the virus entry. Notably, SARS-CoV and SARS-CoV-2 share 90% sequence identity in their S protein S2 subunits (fusion domain, FD) that mediate membrane fusion, and their S1 subunits bind to the human angiotensin-converting enzyme 2 (hACE2) receptor to infect human cells^7,8^. Importantly, recent biophysical and structural studies have shown that the ACE2-binding affinity of the receptor-binding domain (RBD, with 74% homology between the two viruses) in the S1 subunit of SARS-CoV-2 is at least fourfold higher than that of SARS-CoV^9,10^, indicating higher infectivity and transmissibility of SARS-CoV-2. Also, recent cell–cell fusion assays have shown that SARS-CoV-2 S protein is much more efficient in mediating cell–cell fusion than SARS-CoV S protein in the absence of exogenous trypsin, indicating that SARS-CoV-2 has a superior plasma membrane fusion capacity than SARS-CoV^11,12^.

Several reports have recently documented the antibody dynamics in convalescent plasma from COVID-19 patients^13,14^. IgM is consistently detected before IgG, peaking at week 2 and declining after 3–5 weeks post-infection, a time frame that varies from patient to patient. IgG peaks around 3–7 weeks post-infection, and generally remains at a high level for at least 8 weeks. Neutralizing antibodies (NAbs) are induced after 1–2 weeks of infection, peaking at week 3 and then gradually decline^13^. Patients with severe symptoms show significantly higher IgM response and NAb titers compared to mildly ill patients, but the differences in IgG response between the severe and mild cases are modest. Notably, antibodies from SARS-CoV-2-infected patients, but not MERS patients, exhibit cross-reactivity to SARS-CoV^14^.

Passive immunotherapy using convalescent human plasma has been successfully employed to treat infectious diseases, including influenza, Ebola, SARS, MERS, and COVID-19^15–21^. Passive transfer of immune plasma can also prevent the replication of SARS-CoV in a mouse model^22^, confirming the protective nature of SARS-CoV neutralizing antibodies (NAbs).

In this study, we tested the blocking activity of convalescent plasma from COVID-19-recovered subjects by cell–cell fusion assays using SARS-CoV-2-S- or SARS-CoV-S-transfected 293 T as the effector cells and ACE2-expressing 293 T as the target cells. In addition, we characterized the humoral immune responses in COVID-19-recovered subjects. Our results revealed that SARS-CoV-2-S exhibits greater capacity in inducing cell–cell fusion compared to SARS-CoV-S, which is consistent with previous studies^11,12^. Importantly, COVID-19 convalescent plasma with high titers of NAbs can block the cell fusion mediated by either SARS-CoV-2-S/ACE2 or SARS-CoV-S/ACE2 interactions. These findings suggest that COVID-19 convalescent plasma may not only inhibit SARS-CoV-2-S but also cross-neutralize SARS-CoV-S-mediated membrane fusion, supporting its potential role as a preventive and/or therapeutic agent against SARS-CoV-2 as well as other SARS-CoV infections.

## Materials and Methods

### Subjects

The study protocol was approved by the joint Institutional Review Board (IRB) of East Tennessee State University and James H. Quillen VA Medical Center (ETSU/VA IRB, Johnson City, TN), and all experiments in the study were performed in accordance with its relevant guidelines and regulations. Written informed consent was obtained from all participants. The study comprised of two populations: 14 non-hospitalized, mild to moderately symptomatic COVID-19-recovered subjects and 13 control subjects, including 8 healthy subjects (HS), 4 Influenza patients, and 1 HIV patient. All COVID-19 subjects were diagnosed by a positive nuclear acid amplification test (NAAT) and recovered from their symptoms; their blood samples were collected at least 2 weeks after the diagnosis. Blood from healthy subjects was obtained from BioIVT (Gray, TN). The plasma was heat-inactivated at 56 °C for 1 h, aliquoted, and then stored at − 80 °C. The characteristics of these COVID-19 subjects are shown in Table 1.

**Table 1:** The characteristics of COVID-19 patients.

ID	Age	Gender	Severity	Hospitalized (Y/N)	Sampling (Day)	Past Medical History	Anti-S1 IgG (ng/ml)	Inhibition of S/ACE2 Interaction (%)
S1	54	F	Mild	N	74	N/A	280	55.0
S2	54	M	Mild	N	72	N/A	1,799	91.2
S3	51	F	Mild	N	78	N/A	898	87.8
S4	47	F	Moderate	N	75	Asthma	1,778	90.0
S5	20	F	Mild	N	77	N/A	1,033	91.1
S6	30	M	Moderate	N	88	N/A	634	80.0
S7	46	F	Moderate	N	119	Cardiomyopathy	34	15.0
S8	33	M	Mild	N	24	Hypertension, HIV	402	66.0
S9	42	F	Moderate	N	29	N/A	110	33.0
S10	42	M	Moderate	N	32	N/A	55	36.0
S11	42	F	Mild	N	17	Hypertension	1,560	86.6
S21	64	F	Mild to Moderate	N	~ 60	N/A	56	8.7
S23	19	M	Mild to Moderate	N	~ 60	Asthma	–	96.5
S30	38	M	Mild to Moderate	N	~ 60	HIV	–	55.0

### Detection of SARS-CoV-2 specific IgG and IgM antibodies

The SARS-CoV-2 Spike S1-RBD IgG and IgM ELISA Detection kit was used to determine levels of IgG and IgM antibodies in the plasma of COVID-19 and control subjects according to the manufacturer’s instructions (GenScript, Piscataway, NJ). Monoclonal IgG and IgM antibodies specific to SARS-CoV-2 were used as positive controls. The concentration of S1 IgG in the COVID-19 convalescent plasma was quantitated using a high-sensitive SARS-CoV-2 S1 IgG ELISA kit (BioVendor, Asheville, NC).

### Detection of SARS-CoV-2 NAbs

The SARS-CoV-2 Surrogate Virus Neutralization Test kit was used to detect NAbs in COVID-19 and control subjects’ plasma that inhibited SARS-CoV-2 S RBD-ACE2 interactions per the manufacturer’s protocol (GenScript). The NAb levels were calculated based on their inhibition extents according to the following equation: [(1-OD value of samples/OD value of negative control) $\times$ 100%]. A neutralizing antibody against SARS-CoV-2 was used as a positive control.

### Blockade of SARS-CoV-2 S-ACE2-mediated cell–cell fusion by COVID-19 convalescent plasma

Target 293 T cells stably expressing hACE2 (ACE2/293 T, kindly provided by Dr. Hyeryun Choe^23^) were cultured in Dulbecco’s modified Eagle’s medium (DMEM) with 10% FBS in the presence of 1 µg/ml puromycin. Effector 293 T cells were transiently transfected with pAAV-CMV-Luc-IRES-EGFP-SV40 alone (as negative control), or co-transfected with pAAV-CMV-Luc-IRES-EGFP-SV40 and pCDNA3.1-SARS-S or pCDNA3.1-SARS2-S plasmids (Addgene, Watertown, MA). After 48 h of transfection, the cells were detached with 0.25% Trypsin, and incubated with or without 10% plasma from COVID-19 patients or control subjects at 37 °C for 30 min in 10% FBS-DMEM or 80 ng/ml Trypsin-DMEM and then overlaid on 70–80% confluent ACE2/293 T cells. After co-culturing for 4 h and 24 h, cell fusion images were captured with an EVOS FL Image System (Life Technologies, Frederick, MD) and the numbers of the fused cells within at least 4 randomly selected fields were counted.

### Plasmid digestion

The pCDNA3.1-SARS-S plasmid was digested with Xbal 1 and BamH 1, and the pCDNA3.1-SARS2-S plasmid was digested with Nhe 1 and Xhol 1, and resulting DNA was resolved by agarose gel electrophoresis. The images were captured with Chemi Doc MP Imaging System (Bio-Rad, Hercules, CA).

### Western blots

The 293 T/ACE2 and 293 T cells were transfected with CMV-Luc-IRES-EGFP-SV40 and pCDNA3.1-SARS-S or pCDNA3.1-SARS2-S plasmids for cell–cell fusion, harvested, and lysed on ice in RIPA lysis buffer (Boston BioProducts, Ashland, MA) in the presence of cOmplete Protease Inhibitor Cocktail (Sigma-Aldrich, St. Louis, MO). The protein concentrations were measured by Coomassie staining (Bio-Rad). Proteins were separated by 10% SDS-PAGE, transferred to nitrocellulose membranes, which were blocked with 5% non-fat milk, 0.1% Tween-20 in Tris-buffered saline (TBS) and incubated with anti-Myc antibody (Cell Signaling, Danvers, MA), anti-C9 antibody (Bionova, Freemont, CA) (Cell Signaling), or anti-SARS-CoV-2 S RBD antibody (Cell Signaling). After washing, the membranes were incubated with horseradish peroxide (HRP)-conjugated secondary antibodies (Cell Signaling), and the proteins were detected using Amersham ECL Prime Western Blotting Detection Reagent (GE Healthcare BioSciences, Pittsburgh, PA). Protein bands were captured by Chemi Doc MP Imaging System. The membranes were stripped and re-probed with an anti-actin antibody (Cell Signaling) for a loading control.

### Immunofluorescence assays

The 293 T cells transfected with pCDNA3.1-SARS2-S plasmid were used to check S protein expression on the cell surface. The cells were fixed with 2% paraformaldehyde (PFA), and then permeabilized with 0.1% Triton X-100/3% BSA in PBS. After blocking with 3% BSA in PBS for 1 h at RT, the cells were incubated with 1:100 diluted rabbit anti-SARS-CoV-2 S RBD antibody (Invitrogen, Waltham, MA) overnight at 4 °C. After washing, the cells were incubated with Alexa 555-conjugated anti-rabbit IgG antibody (Invitrogen) for 1 h at RT. The DAPI was used for counterstaining nuclei/DNA post-secondary washing. The fluorescent images were captured with an EVOS FL Image System.

### SARS-CoV-2-S _Δ19_ Pseudotyped Luciferase-EGFP lentivirus

The plasmids pHIVNLGagPol, pCCNanoLuc2AEGFP, and pSARS-CoV-2-S_Δ19_ were kindly provided by Dr. Paul D. Bieniasz (The Rockefeller University, New York, NY) To generate SARS-CoV-2-S pseudotyped luciferase-EGFP lentivirus, 293 T cells were co-transfected with pHIVNLGagPol, pCCNanoLuc2AEGFP, and pSARS-CoV-2-S_Δ19_ using PEI (Polyscience, Warrington, PA) as described previously^24^. 48 h after transfection, the supernatant was harvested, filtered with a 0.45 µm syringe filter, aliquoted, and stored at − 80 °C for infection of target cells.

### Neutralization assay with pseudovirus (PsV)

The 293 T/ACE2 target cells were seeded in a 96-well plate (10^4^ cells in 100 µl medium per well) and cultured overnight in a CO_2_ incubator at 37 °C. The heat-inactivated plasma from convalescent COVID-19 patients was serially diluted (fivefold) with DMEM/10%FBS. Approximately 30 µl of undiluted or diluted plasma were mixed with 20 µl of PsV and incubated for 30 min at 37 °C, then added to the cultured 293 T/ACE2 cells in the presence of 10 µg/µl of polybrene for infection. The fluorescent images were captured at 72 h post-infection with an EVOS FL Image System. The infected 293 T/ACE2 cells were lysed and the luciferase activities were measured using Nano-Glo Luciferase Assay System (Promega, Madison, WI) and a BioTek SYNERGY H1 microplate reader. The titers of NAbs were calculated as 50% inhibitory concentration (IC50), which is the plasma dilution factor resulting in a 50% reduction in luciferase relative light unit (RLU) compared with the control.

### Statistical analysis

The data were analyzed using Prism 6.01 software and are presented as mean ± standard error of the mean (SEM). Student’s *t*-tests were used to compare the means of two independent groups with equal variances. The magnitude of correlation was analyzed with Pearson’s correlation coefficient (parametric approach). *P*-values of < 0.05 were considered statistically significant.

## Results

### Determining SARS-CoV-2 specific antibodies in COVID-19 convalescent plasma

**Figure 1:**
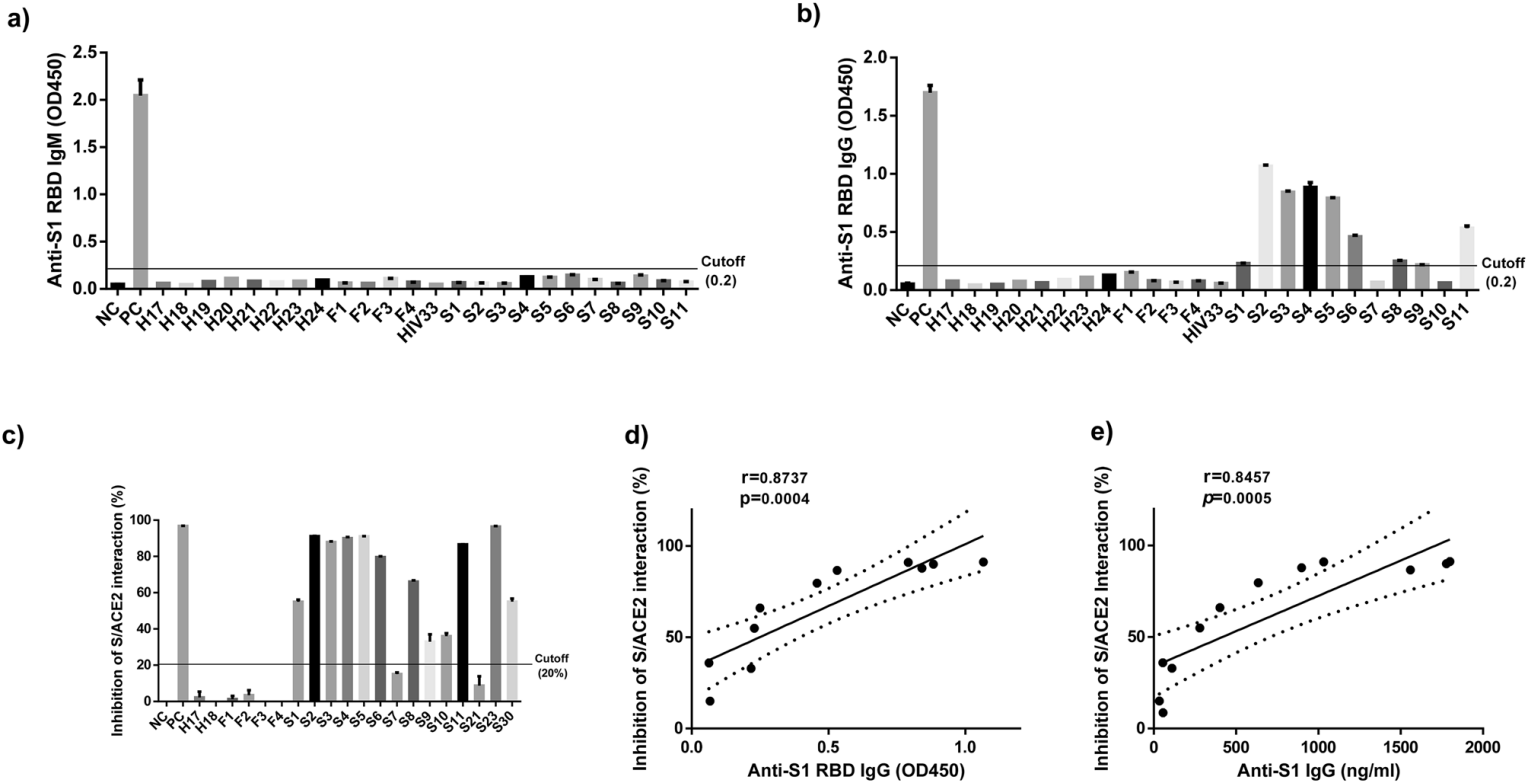
Detection of SARS-CoV-2 specific antibodies in the plasma of COVID-19-recovered subjects. (**a**–**b**) The IgM and IgG antibodies against SARS-CoV-2-S1 RBD were detected in the plasma of COVID-19-recovered subjects and control subjects by ELISA. (**c**) The NAbs that block the interactions of SARS-CoV-2-S1 RBD and hACE2 were determined by an ELISA Test kit. (**d**–**e**) The correlation between the inhibition extent of NAbs and OD450 values of anti-S1 RBD as well as anti-S1 IgG antibodies. All the graphs in 1 were created by Prism 6.01 software (https://www.graphpad.com/).

To characterize COVID-19 humoral immune responses, we collected blood samples from COVID-19-recovered subjects and assayed for the presence of specific IgM and IgG antibodies in plasma (1:100 diluted) by ELISA Kits, which use SARS-CoV-2-S1 RBD as a capture antigen. As shown in Fig. 1a, all tested subjects were negative for anti-RBD IgM antibody, including COVID-19-recovered subjects and control individuals. Amongst the eleven COVID-19 subjects studied, six (S2, S3, S4, S5, S6, and S11) remained positive for anti-RBD IgG antibody, three (S1, S8, and S9) were marginally positive or cutoff (OD 450: 0.2), and two (S7–119 days after diagnosis and S10–32 days after diagnosis) were IgG negative (Fig. 1b). All control subjects, including eight healthy subjects (H), four Influenza subjects (F), and one HIV subject (HIV), were tested negative for the SARS-CoV-2 S1 RBD IgG antibody. These results suggest that SARS-CoV-2 S1 RBD IgG antibodies in the plasma of some COVID-19 patients are maintained at very low levels or diminished quickly after recovery. To ensure virus-specific antibodies against other epitopes on the SARS-CoV-2 S1 subunit, which contains N-term domain (NTB) and RBD, a high-sensitive SARS-CoV-2 S1 IgG ELISA kit (BioVendor, Asheville, NC) was used for the quantitative detection of anti-S1 IgG titers in the patient's plasma. Notably, the titers of anti-S1 IgG antibody (Table 1) were found compatible with the OD450 values of anti-RBD IgG antibody (Fig. 1b).

To measure protective NAbs, COVID-19 convalescent plasma was diluted (1:10) and incubated with HRP-conjugated, recombinant SARS-CoV-2-S1 RBD for 30 min at 37 °C prior to adding to an ACE2 pre-coated ELISA plates. The inhibition extents of S and ACE2 interactions were calculated as described in the Materials and Methods. As shown in Fig. 1c and Table 1, seven of the COVID-19 subjects (S2, S3, S4, S5, S6, S11 and S23) exhibited inhibition extents greater than 80–90%; five COVID-19 subjects (S1, S8, S9, S10 and S30) showed 30–70% inhibition extents; two COVID-19 subjects (S7 and S21) showed inhibition extents lower than the 20% cutoff which suggesting no detectable SARS-CoV-2-S1 RBD NAbs. All negative controls (four Influenza patients and two healthy subjects) showed undetectable NAbs to SARS-CoV-2-S1 RBD. Importantly, the inhibition extents positively correlated with the levels of SARS-CoV-2-S1 anti-RBD IgG antibodies (r = 0.8737, *p* = 0.0004) (Fig. 1d) as well as the anti-S1 IgG antibodies (r = 0.8457, *p* = 0.0005) in these COVID-19 patients (Fig. 1e), analyzed by the Pearson Correlation. These results indicate that most of the COVID-19-recovered patients have high titers of NAbs to SARS-CoV-2-S1 as well as RBD epitopes in their convalescent plasma.

### Blockade of SARS-CoV-2-S/293 T or SARS-CoV-S/293 T and ACE2/293 T-mediated cell–cell fusions using COVID-19 convalescent plasma

The S proteins of SARS-CoV-2 and SARS-CoV have an RBD in S1 and an FD in S2 working in synergy to bind to the ACE2 receptor on target cells and induce cell membrane fusion for viral entry^11,12,25^. To establish an assay for measuring SARS-CoV-2-S-mediated cell–cell fusion, we employed 293 T cells expressing both SARS-CoV-2-S and enhanced green fluorescent protein (EGFP) as effector cells and 293 T cells stably expressing the human ACE2 receptor (ACE2/293 T) as target cells.

**Figure 2:**
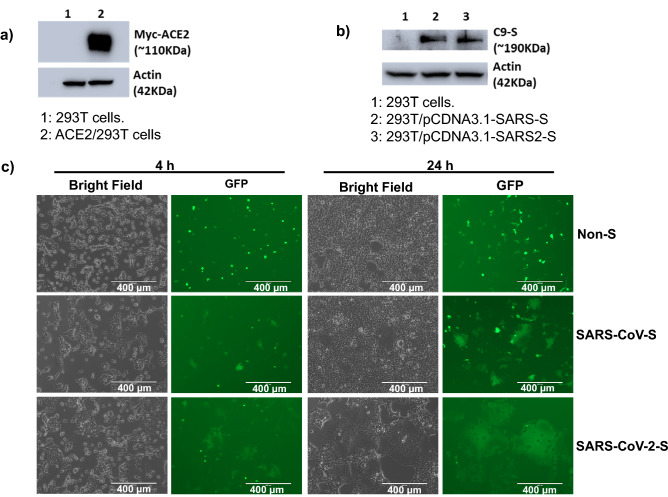
Establishment of SARS-CoV-2-S-mediated cell–cell fusion system. (**a**) Western blot analysis of S protein expression in 293 T cells transfected with pCDNA3.1-SARS-S and pCDNA3.1-SARS2-S. The two blot images derived from different gels which were run using the same samples. (**b**) Western blot analysis of ACE2/293 T cells that express ACE2 protein. The two blot images derived from different gels which were run using the same samples. The full blot images for a and b are included in the supplementary figures. (**c**) Imaging of SARS-CoV-S/293 T and SARS-CoV-2-S/293 T cell fusion with ACE2/293 T cells at 4 h and 24 h. Non-S-transfected cells serve as a negative control. Scale bar equals 400 µm in all figures.

**Figure 3:**
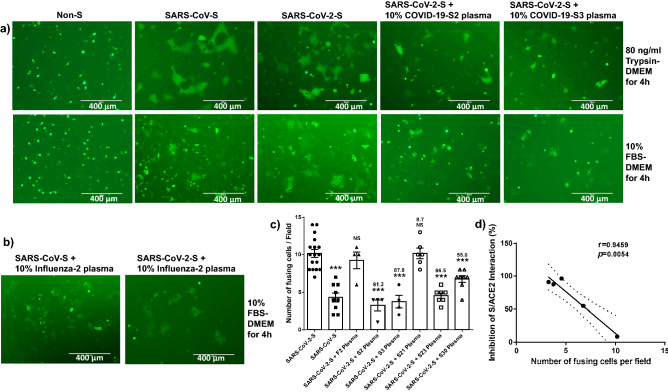
Convalescent plasma from COVID-19-recovered subjects inhibits cell–cell fusion mediated by the S-ACE2 interactions. (**a**) The effector cells: non-S-transfected 293 T (EGFP-alone), SARS-CoV-S-transfected 293 T, SARS-CoV-2-S-transfected 293 T, or SARS-CoV-2-S-transfected 293 T cells were incubated with 10% COVID-19 S2 plasma. SARS-CoV-2-S-transfected 293 T cells with 10% COVID-19 S3 plasma were incubated with the ACE2/293 T target cells for 4 h with 80 ng/ml of trypsin-DMEM (upper panel) or 10% FBS-DMEM (lower panel). (**b**) Plasma from Influenza-recovered subject cannot inhibit cell–cell fusion mediated by the S-ACE2 interactions. (**c**) The number of cell–cell fusions induced by SARS-CoV-S, SARS-CoV-2-S, and SARS-CoV-2-S following pre-incubated with 10% COVID-19 S2, S3, S21, S23, S30 or F2 plasma are shown (*P < 0.05, **P < 0.01, and ***P < 0.001, respectively; the numbers on top of the bars represent the inhibition extent percentiles of S/ACE2 interaction of the convalescent plasma). d) The correlation between the inhibition extent of S1/ACE2 interaction and the number of fusing cells per field were shown. The graphs in c and d were created by Prism 6.01 software (https://www.graphpad.com/).

Figure S1a shows the results of the restriction enzyme digestion of the pCDNA3.1/SARS-CoV-2-S and pCDNA3.1/SARS-CoV-S plasmids used for cell transfection, both of which contain the S gene. Figure 2a shows similar expression levels of SARS-CoV S and SARS-CoV-2 S protein in the plasmid-transfected effector cells. Western blot analysis showed that the stably transfected ACE2/293 T cells expressed the Myc-ACE2 protein (Fig. 2b), and immunofluorescence assays showed cell surface expression of the S protein in pCDNA3.1/SARS-CoV-2-S transfected 293 T cells (Fig. S1b). Notably, when the effector cells and the target cells were co-cultured at 37ºC for 4 h and 24 h, the two types of cells started to fuse at 4 h and the fused cells exhibited a much larger size and multiple nuclei compared to the unfused cells. These changes were more significant at 24 h, resulting in hundreds of cells fused as one large syncytium with multiple nuclei that could be easily seen under both light and fluorescence microscopy (Fig. 2c), which is similar with syncytium formation induced by SARS-CoV-2 infection ^11,26^. Also, the fluorescence intensity in the fused cells became weaker as a result of EGFP diffusion from the effector cells to multiple target cells. The cell fusions were observed in both SARS-CoV and SARS-CoV-2 groups, whereas those cells transfected with EGFP only without the S protein did not elicit such an effect, confirming that CoV S-ACE2 engagement is essential for viral fusion and entry. Consistent with a previous study showing that SARS-CoV-2 S protein binds to ACE2 with a higher affinity than SARS-CoV S protein^9^, our results showed that the SARS-CoV-2 group exhibited a greater number and larger size of fused cells compared to the SARS-CoV group (Figs. 2c and 3). These results support the notion that SARS-CoV-2 S has a higher ACE2 binding affinity (related to S1 RBD) and/or fusion capacity (related to S2 FD) that mediates viral fusion and entry into the target cells compared to SARS-CoV S^25^.

To determine whether the plasma of COVID-19 patients can block S protein-mediated cell–cell fusion, we incubated the effector cells with COVID-19 convalescent plasma with different titers of NAbs (S2, S3, S21, S23 and S30) at 37 °C for 30 min and then washed the cells prior to co-culturing with the ACE2/293 T target cells. This step allows the S protein expressed on the effector cells to sufficiently bind to the S protein-recognizing antibodies present in the plasma. We first performed cell–cell fusion assays using 80 ng/ml of Trypsin-DMEM, which facilitates plasma membrane fusion^11,12,25^. We found that cell fusion occurred in both SARS-CoV-S and SARS-CoV-2-S groups compared to non-S, EGFP-transfected cells at 4 h after the effector cells were overlaid on top of the target cells under this condition. However, not only fewer fusing cells were observed, but also the sizes of fused cells were much smaller in the groups of SARS-CoV-2-S/293 T effector cells pre-incubated with 10% or 20% convalescent plasma from COVID-19 patients compared to that without patient plasma pre-incubation (Fig. 3a, upper panel).

We also employed a culture condition with 10% fetal bovine plasma (FBS, without trypsin)-DMEM for cell–cell fusion assays and obtained the same results (Fig. 3a, lower panel), except that trypsin appeared to be essential for SARS-CoV-S-mediated membrane fusion (at least at the early, 4 h incubation phase), whereas SARS-CoV-2 did not require exogenous trypsin for efficient cell fusion (compare to Fig. 3a, upper panel). Counting of fused cells per field in at least four randomly selected fields revealed a remarkably higher number of cell–cell fusions in the SARS-CoV-2 group than the SARS-CoV group, and the SARS-CoV-2 S-mediated cell–cell fusions were significantly reduced by convalescent plasma with high titers of NAbs (S2, S3, S23 and S30), but not by S21 convalescent plasma with undetectable NAbs (Figs. 3c and S2a). Further Pearson Correlation analysis showed that the numbers of fusing cells per field negatively correlated with inhibition extents of S/ACE2 interaction (Fig. 3d), indicating that the efficiency of blocking cell–cell fusions is closely associated with the convalescent plasma NAb titers. As negative controls, plasma from Influenza-2 patient or HS-19 was also tested using the same approach, but did not elicit significant inhibition on cell–cell fusion mediated by the SARS-CoV-2 S-ACE2 interactions (Figs. 2a, 3b, and c). Western blots were performed using whole cell lysates from the cell–cell fusion samples with anti-SARS-CoV-2 S Protein (RBD) antibody, which recognizes the full-length S protein and cleaved S1 protein. Notably, the cleaved S1 bands were much weaker compared to full-length S bands, and there were no significant differences in S1 protein levels among different samples (Fig. S2b). Taken together, these results demonstrate that SARS-CoV-2-S protein could effectively mediate cell–cell fusion in the absence of an exogenous proteolytic enzyme (e.g., trypsin), and the COVID-19 convalescent plasma could neutralize SARS-CoV-2 S-mediated membrane fusion and virus entry.

### Plasma from convalescent COVID-19 patients specifically inhibit SARS-CoV-2 infection

The pSARS-CoV-2-S_Δ19_ plasmid expresses a SARS-CoV-2 S mutant, which is truncated 19 amino acids from the C-terminus to improve the assembly of lentivirus expressing S protein. Based on a previous study, PsV made with truncated S protein generates about tenfold higher titers of infectious particles than those made with a full-length SARS-CoV-2 S protein^24^. Thus, this PsV expressing SARS-CoV-2-S_Δ19_ plasmid was used for our neutralization assays.

**Figure 4:**
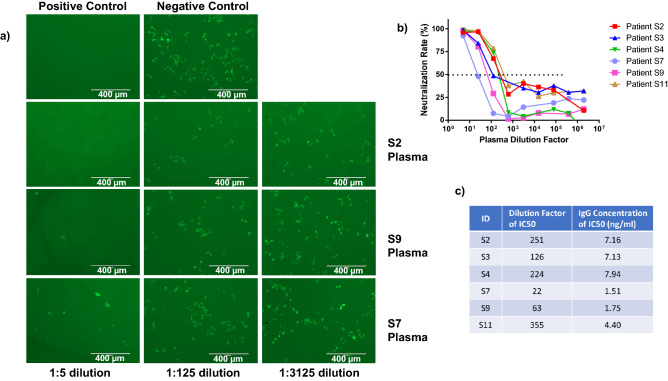
Inhibition of SARS-CoV-2 infection by COVID-19 convalescent plasma. (**a**) Representative fluorescent images showing inhibition of SARS-CoV-2 infection by convalescent patient's plasma. Negative and positive controls of PsV infection into 293 T/ACE2 target cells are shown. (**b**) Neutralization assays showing that COVID-19 convalescent plasma inhibits SARS-CoV-2 S PsV infection. (**c**) The dilution factors of patient plasma and their corresponding concentrations of anti-S1 IgG antibodies are shown. The graph in b was created by Prism 6.01 software (https://www.graphpad.com/).

Since the PsV carried both luciferase and EGFP genes, the fluorescent images were captured before performing the luciferase assays, and representative EGFP images are shown in Fig. 4a. Notably, using convalescent plasma from COVID-19-recovered subjects with high (S2), medium (S9), and low IgG/NAb antibodies (Fig. 1b and c), we observed more EGFP^+^ cells in samples with more dilution and/or with lower IgG level (Fig. 4a), which indicates that PsV infection inhibitory capacity was reduced by the dilution and lower IgG level of convalescent plasma. Our neutralization assays showed that all six patients’ plasma exhibited a concentration-dependent inhibition of SARS-CoV-2 S PsV infection. S7 plasma (with the lowest IgG/NAb level) showed the weakest inhibition of viral infection, whereas S2, S4 and S11 plasma (with relatively higher IgG/NAb levels) showed a relatively stronger inhibition than other patient plasma (Fig. 4b). These results are consistent with the titers of S1 IgG neutralizing antibodies (Table 1), suggesting that a higher titer of IgG NAbs has a stronger inhibition for SARS-CoV-2 infection. According to Fig. 4b**,** the plasma dilution factors and the amounts of S1 IgG antibodies in the plasma were considered and a calculation was made on the basis that 1.51–7.16 ng/ml of S1 IgG antibodies resulted in a 50% reduction in SARS-CoV-2 infection (Fig. 4c).

## Discussion

Despite the poorly defined pathogenesis of COVID-19, the majority of SARS-CoV-2-infected individuals spontaneously recover, suggesting that host immunity is naturally induced in COVID-19-recovered subjects. An explicit study of COVID-19, particularly of host immunity to this viral infection in recovered subjects, will lay a foundation for a rational design of therapeutics and/or vaccines against future outbreaks. In this study, we examined the SARS-CoV-2 specific IgM and IgG antibodies, and found that IgM antibodies against the S protein RBD domain were negative for all COVID-19 subjects, even for one subject (S11) whose blood was collected 17 days after the onset of symptoms. These results suggest that the IgM antibody against SARS-CoV-2-S1 RBD diminishes quite early, or that it may never achieve a seroconversion for IgM antibody, against the RBD epitope during viral infection. Amongst the 11 COVID-19 subjects tested for IgG antibodies, 6 subjects remained positive, 3 subjects were borderline, and 2 were negative. These results indicate that SARS-CoV-2 RBD-specific antibodies may wane quickly after recovery in some patients and are not reliable for assessing humoral immune responses or herd immunity.

Virus neutralization is the reduction of virus infectivity by an antibody^27^. NAbs protect host cells against viral infection by various neutralization mechanisms. For example, NAbs can bind with virions and prevent them from attaching to the target cells. Also, NAbs can block the receptor necessary for the interactions between the virions and target cells. Moreover, NAbs can inhibit viral entrance to the target cells at different stages, such as coreceptor engagement, endocytosis, membrane fusion, or viral penetration^28^.

As the primary target for NAbs, the RBD of SARS-CoV-2 S1 subunit is a major focus for the development of NAbs against SARS-CoV-2. Many NAbs targeting the RBD have been shown to inhibit the association between the S protein and ACE2^29–36^. Sequence comparisons of MERS-CoV strains have shown that the RBD of the S protein has very high natural mutation rate, indicating that individually applied RBD-targeting antibodies might induce resistance mutations in the virus^37^. Some antibodies targeting the N-terminal domain (NTD) of the SARS-CoV-2 S1 subunit exhibit high neutralization potency to SARS-CoV-2^38,39^. The heptad repeat loops (HR1 and HR2) of the S2 subunit, required for membrane fusion, might be another good target. The NAbs that target the S2 subunit have rarely been reported. The 1A9 antibody is the only known monoclonal antibody that binds the HR2 domain on S2 subunit of SARS-CoV, and it can block HR1-HR2 interaction and thus inhibit S2-mediated membrane fusion^40^. S21P2 is a SARS-CoV-2 peptide that covers part of the fusion peptide (FP) of S2 subunit. One study has reported that sera depleted of antibodies that target peptide S21P2 can significantly reduce the ability to neutralize SARS-CoV-2 pseudovirus infection compared with the non-depleted sera control^41^.

Interaction between SARS-CoV-2 S protein with the cellular receptor is the first step for viral entry and infection. Specifically, after the RBD in the S1 subunit of SARS-CoV-2 S protein on the virion binds to the ACE2 receptor on target cells, the FDs in the S2 subunit of the S protein undergo conformational changes and interact with each other, thus bringing the viral and cellular membranes into close proximity and allowing for the fusion peptide be inserted into the host target cell membrane for fusion, thus facilitating viral infection as well as transmission^42^. In this study, we demonstrate that SARS-CoV-2 has a greater capacity to induce cell–cell fusion than SARS-CoV. Coronavirus entry into target cells can be achieved via two routes: early plasma membrane pathway and late endosome pathway^43^. It has been reported that protease cleavage is required for activation of the fusion potential of CoV-S proteins^44–46^ and that the availability of these proteases, including furin, trypsin, cathepsins, transmembrane protease serine protease-2 (TMPRSS2), and human airway trypsin-like protease on the target cells determines whether CoVs enter the cells through the plasma membrane or endocytosis^47–54^. In the absence of exogenous or membrane-bound proteases, coronavirus can be internalized via the endosome pathway^55,56^. In this study, we discovered that SARS-CoV-2 can efficiently induce cell–cell fusion without the need to an exogenous proteolytic enzyme (e.g., trypsin), suggesting that SARS-CoV-2 may utilize the plasma membrane fusion pathway to enter the host cells. Previous studies showed that TMPRSS2 (a serine protease) plays an important role in the cleavage and activation of SARS-CoV S protein that is required for membrane fusion and host cell entry^57–59^. In addition, recent studies have demonstrated that SARS-CoV-2 also utilizes TMPRSS2 for SARS-CoV-2 S protein priming and S protein-driven cell entry into human lung epithelium and small intestinal enterocytes^60,61^. TMPRSS2 may also thwart the antiviral effect of Interferon-induced transmembrane proteins (IFITMs) that block virus entry by inhibiting S protein-mediated fusion^26^. Notably, Camostat mesylate, a clinically approved TMPRSS2 inhibitor, partially blocked SARS-CoV-2 S protein-driven entry into target cells. Complete inhibition of TMPRSS2 was achieved by the simultaneous use of camostat mesylate and E-64d (an inhibitor of cathepsin B/L), indicating that both TMPRSS2 and cathepsin B/L can be used for SARS-CoV-2 S protein priming^60^. Thus, the research to develop specific inhibitors for SARS-CoV-2 S may reveal potential targets for blocking the viral life-cycle and infection or transmission.

Recently, the FDA has approved convalescent plasma as a potential treatment for COVID-19^62^. Our findings support this approval by the evidence that the COVID-19 convalescent plasma could recognize and bind to the SARS-CoV-2 S protein expressed on the effector cells, resulting in inhibition of S protein binding to the ACE2 receptor on the target cells and thus blockade of cell–cell fusion. Also, we observed cross-reactivity between SARS-CoV-2 plasma and SARS-CoV, suggesting that COVID-19 convalescent plasma may not only neutralize SARS-CoV-2 S-mediated but also SARS-CoV S-mediated membrane fusion and virus entry. Other clinical parameters, such as the time of convalescent plasma delivery following viral infection, the concentration of neutralizing antibody within the donated convalescent plasma, and the presence or absence of an existing host humoral immune response, may complicate the interpretation of clinical trial data. However, our study presents the potential paradigm for using COVID-19 convalescent plasma as a preventive and therapeutic agent against SARS-CoV-2 infection.

## Supplementary Information

Supplementary Information

## Data Availability

The datasets generated and analyzed during the course of this study are available from the corresponding author upon reasonable request. The data sharing policies will be followed per NIH and VA guidelines.
