# Supplementary material for: Blockade of SARS-CoV-2 spike protein-mediated cell–cell fusion using COVID-19 convalescent plasma
Source: Sci Rep. 2021 Mar 10;11:5558. doi: 10.1038/s41598-021-84840-3 (PMC7946952; doi:10.1038/s41598-021-84840-3)
Supplement: Supplementary file 1 — Supplementary Information [file 41598_2021_84840_MOESM1_ESM.pdf]

Supplementary

Blockade of SARS-CoV-2 spike protein mediated cell-cell fusion using COVID-19 convalescent plasma

Ling Wang[1,2], Juan Zhao[1,2], Lam N.T. Nguyen[1,2], James L. Adkins[1], Madison Schank[1,2], Sushant Khanal[1,2], Lam N. Nguyen[1,2], Xindi Dang[1,2], Dechao Cao[1,2], Bal Krishna Chand Thakuri[1,2], Zeyuan Lu[1], Jinyu Zhang[1,2], Yi Zhang[1], Xiao Y. Wu[1,2], Mohamed El Gazzar[1,2], Shunbin Ning[1,2], Jonathan P. Moorman[1,2,3], and **Zhi Q. Yao[1,2,3]

[1]Center of Excellence for Inflammation, Infectious Disease and Immunity, James H. Quillen College of Medicine, East Tennessee State University, Johnson City, Tennessee 37614

[2]Division of Infectious, Inflammatory and Immunologic Diseases, Department of Internal Medicine, Quillen College of Medicine, ETSU, Johnson City, Tennessee 37614

[3]Hepatitis (HCV/HBV/HIV) Program, James H. Quillen VA Medical Center, Department of Veterans Affairs, Johnson City, Tennessee 37614

Running title: COVID-19 convalescent plasma blocking cell-cell fusion

**Corresponding author: Zhi Q. Yao, M.D., Ph.D.

Director, Center of Excellence for HIV/AIDS Care,

Quillen College of Medicine, East Tennessee State University,

Johnson City, TN 37614

Tel: 423-439-8029; Fax: 423-439-7010; E-mail: yao@etsu.edu

Supplementary Figure S1

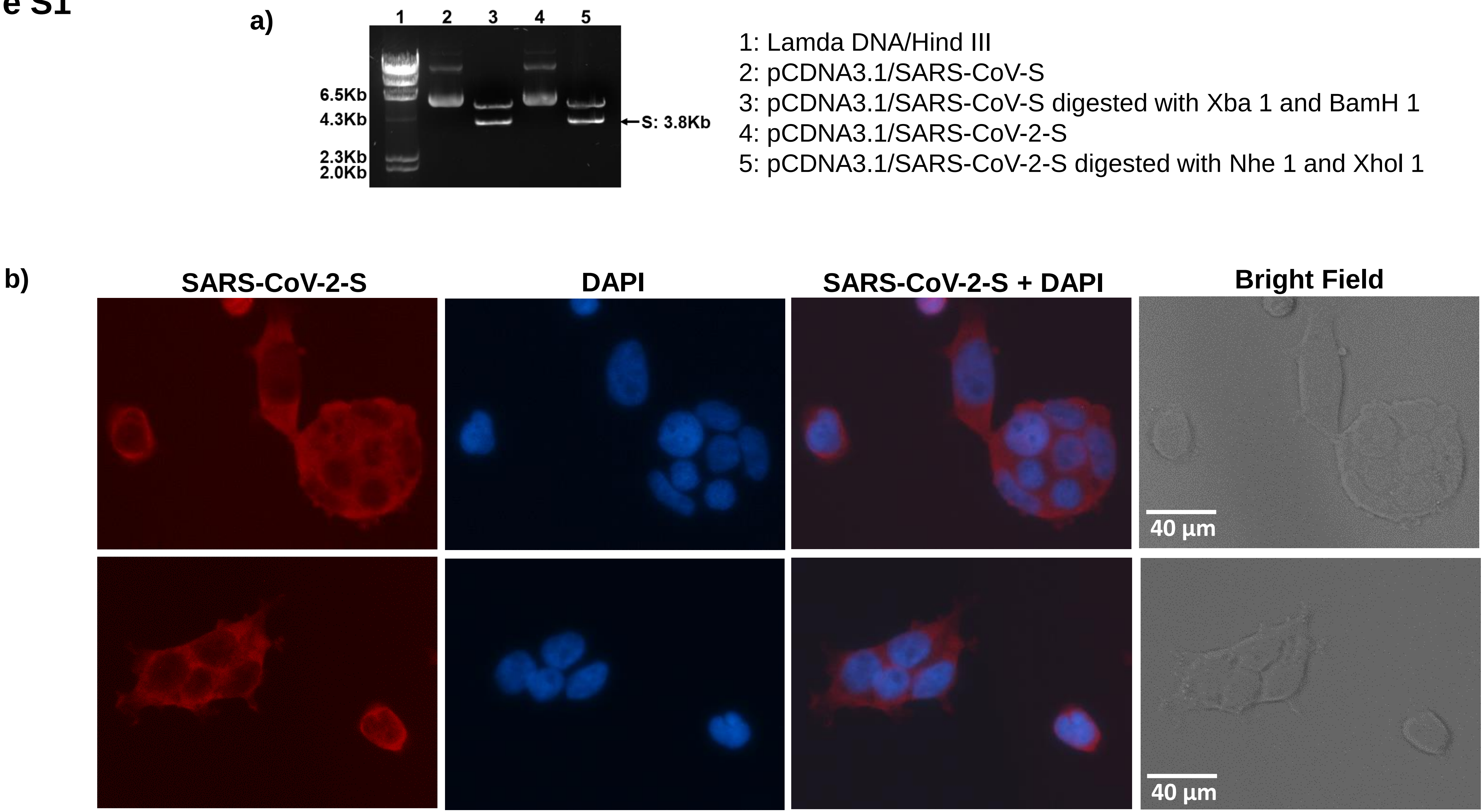

Supplementary Figure S2

a)

Non-S + 10% FBS/DMEM | SARS-CoV-2-S + 10% FBS/DMEM | SARS-CoV-2-S + 10% HS19 plasma

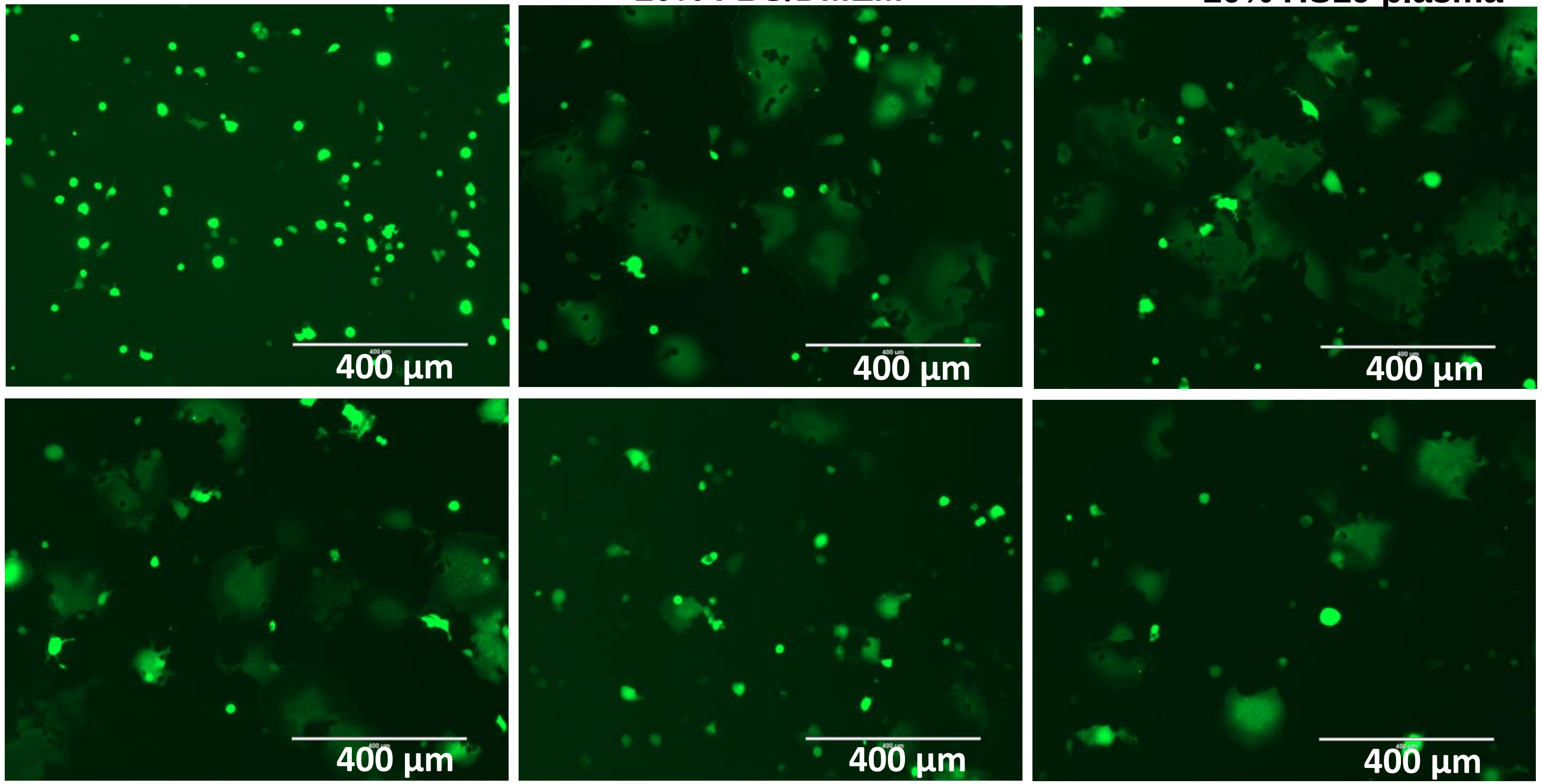

SARS-CoV-2-S + 10% COVID-19-S21 plasma | SARS-CoV-2-S + 10% COVID-19-S23 plasma | SARS-CoV-2-S + 10% COVID-19-S30 plasma

b)

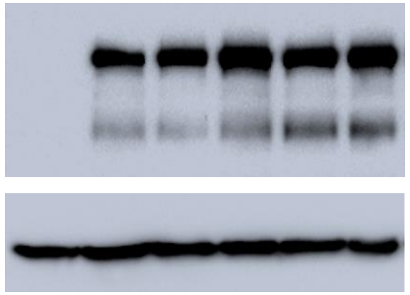

1: 293T transfected with GFP plasmid + 293T/ACE2 cells
2: 293T transfected with GFP and pCDNA3.1-SARS2-S plasmids + 293T/ACE2 cells
3: 293T transfected with GFP and pCDNA3.1-SARS2-S plasmids + 293T/ACE2 cells + 10% HS19 plasma
4: 293T transfected with GFP and pCDNA3.1-SARS2-S plasmids + 293T/ACE2 cells + 10% S21 plasma
5: 293T transfected with GFP and pCDNA3.1-SARS2-S plasmids + 293T/ACE2 cells + 10% S23 plasma
6: 293T transfected with GFP and pCDNA3.1-SARS2-S plasmids + 293T/ACE2 cells + 10% S30 plasma

Supplementary Figure S3

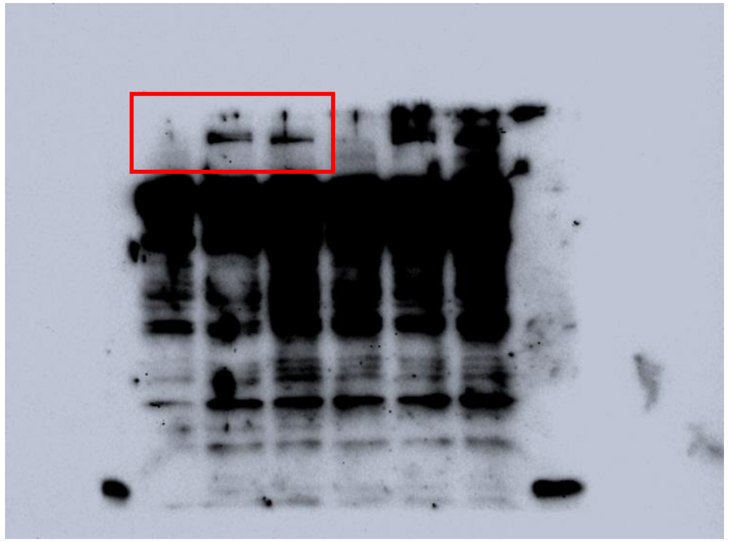

Fig. 2a: C9-S

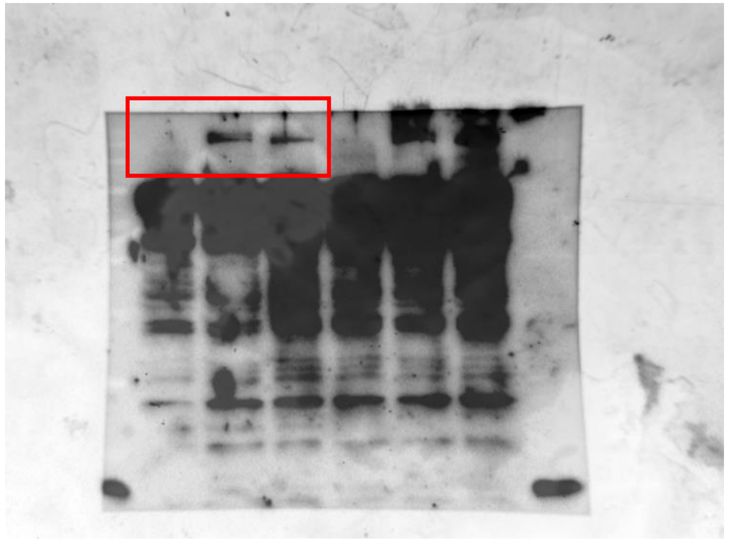

Fig. 2a: C9-S
Merged with Bright Field Image

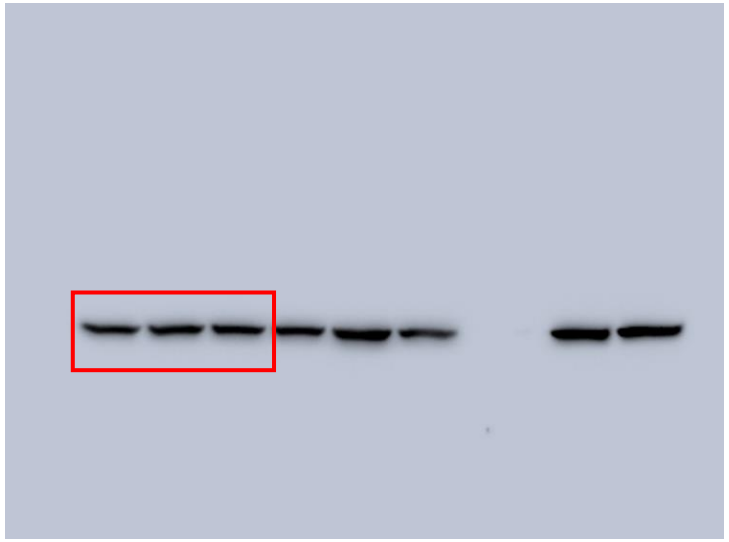

Fig. 2a: Actin

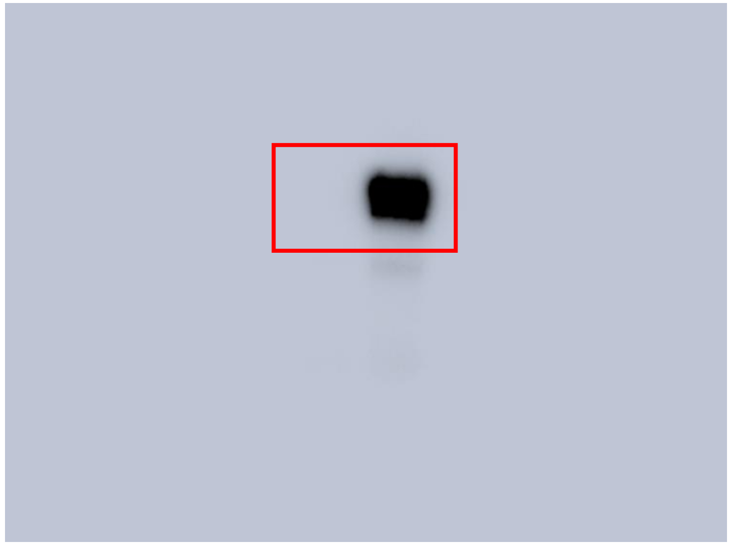

Fig. 2b: Myc-ACE2

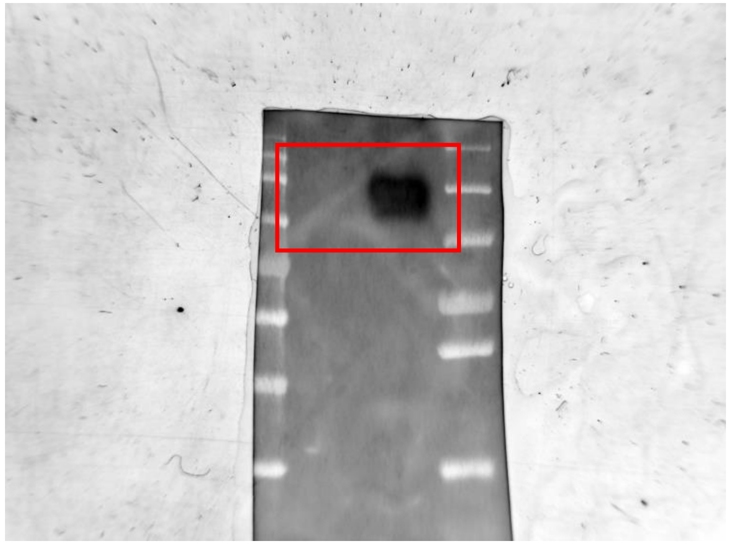

Fig. 2b: Myc-ACE2
Merged with Bright Field Image

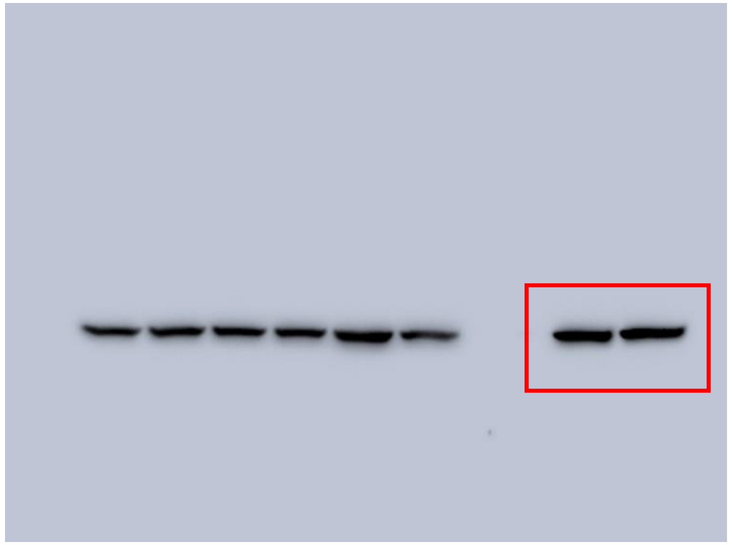

Fig. 2b: Actin

Supplementary Figure S4

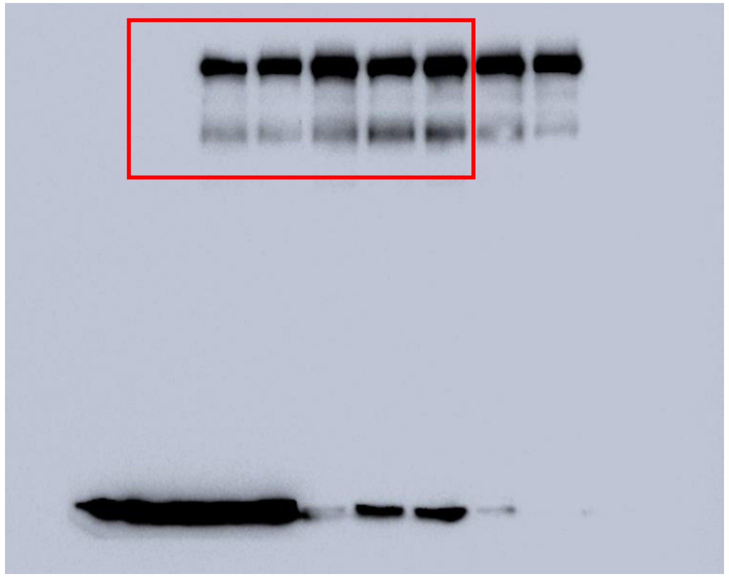

Fig. S2b: Full-length S and S1

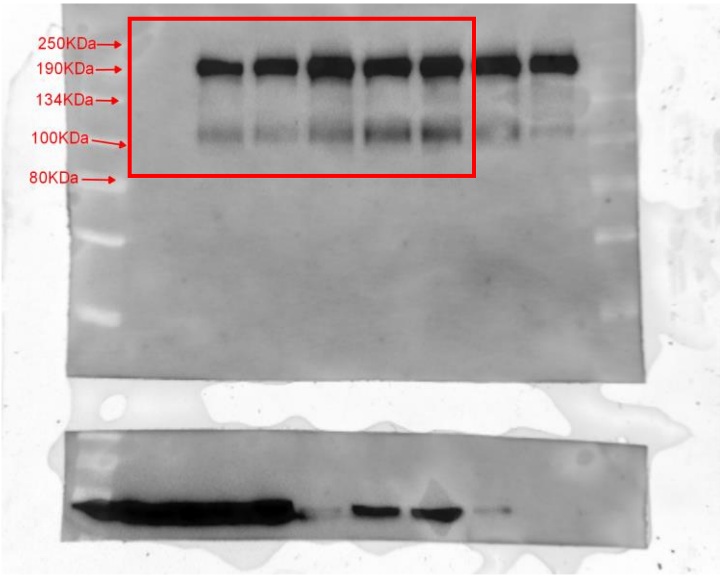

Fig. S2b: Full-length S and S1
Merged with Bright Field Image

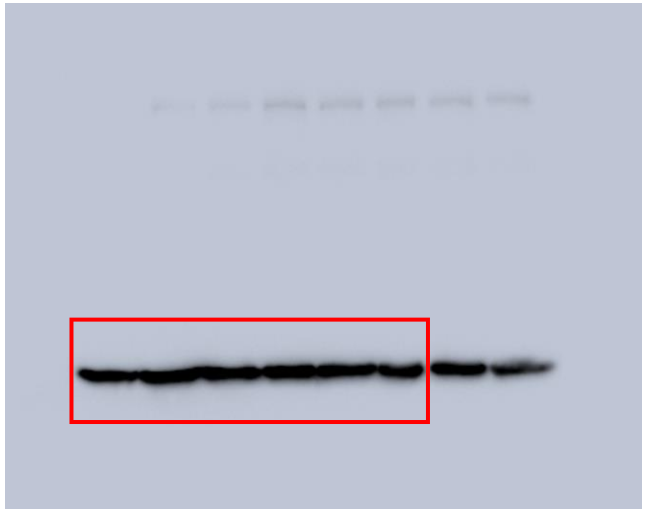

Fig. S2b: Actin

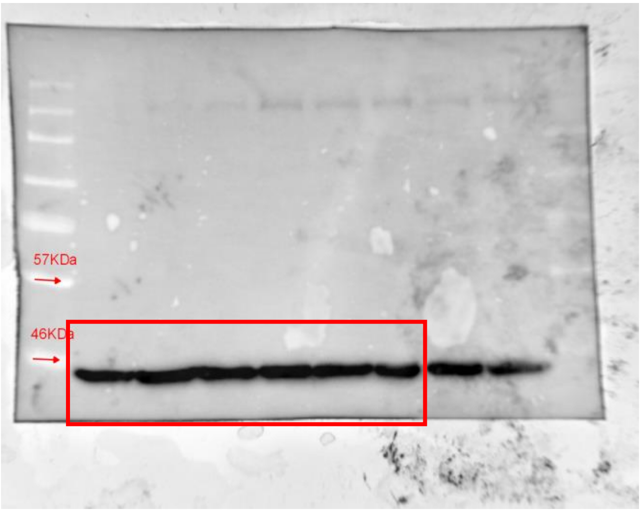

Fig. S2b: Actin
Merged with Bright Field Image

Supplementary Figure Legends

Figure S1. a) Restriction enzyme digestion of SARS-CoV-2-S and SARS-CoV-S plasmids. **b)** Immunofluorescence assays to check SARS-CoV-2-S expression on cell surface.

Figure S2. a) The COVID-19 convalescent plasma with high titers of NAbs blocks SARS-CoV-2-S-mediated cell-cell fusions. **b)** Western blot analysis of expression of full-length S protein and S1 protein (Cleaved band). The two blot images were cropped from the same gel.

Figure S3. Original Western blot images for **Figure 2a** and **2b**. The images for **Figure 2a**-actin and **Figure 2b**-actin were from one same original full-length membrane. Because there were very dominant actin bands without any other nonspecific bands on the membrane, we did not capture bright field image for the membrane.

Figure S4. Original Western blot images for **Figure S2b**.
